# Maximum Entropy Approach to Reliability of Multi-Component Systems with Non-Repairable or Repairable Components

**DOI:** 10.3390/e23030348

**Published:** 2021-03-15

**Authors:** Yi-Mu Du, Jin-Fu Chen, Xuefei Guan, C. P. Sun

**Affiliations:** 1 Graduate School of China Academy of Engineering Physics, Beijing 100193, China; ymdu@gscaep.ac.cn (Y.-M.D.); chenjinfu@csrc.ac.cn (J.-F.C.); 2 Beijing Computational Science Research Center, Beijing 100193, China

**Keywords:** statistical inference, maximum entropy principle, hazard rate function, complex network

## Abstract

The degradation and recovery processes are multi-scale phenomena in many physical, engineering, biological, and social systems, and determine the aging of the entire system. Therefore, understanding the interplay between the two processes at the component level is the key to evaluate the reliability of the system. Based on the principle of maximum entropy, an approach is proposed to model and infer the processes at the component level, and is applied to repairable and non-repairable systems. By incorporating the reliability block diagram, this approach allows for integrating the information of network connectivity and statistical moments to infer the hazard or recovery rates of the degradation or recovery processes. The overall approach is demonstrated with numerical examples.

## 1. Introduction

Degradation processes are ubiquitous in many physical, engineering, biological, and social systems. Modeling the degradation is crucial for lifetime prediction and has drawn increasing attention in the field of reliability and risk analysis [1]. Reliable and accurate lifetime prediction remains a great challenge due to the time-varying and stochastic nature of degradation processes.

In reliability theory, the hazard rate function characterizes the failure probability in the degradation processes, and determines the probability distribution of the lifetime. To estimate the hazard rate function, the lifetime distribution is usually presumed in a certain form, and is fitted with the lifetime testing data. Alternatively, with a large number of lifetime data, an empirical curve can be directly established by interpolation. Both methods require sufficient samples of data to assure the accuracy and reliability of the results. For high reliability-demanding systems or parts, the sample size is usually small. To alleviate the difficulty, the previous study [2] proposed a method based on the maximum entropy principle (MaxEnt) [3,4,5] to estimate the hazard rate function and the lifetime distribution with limited lifetime testing data of the whole system.

However, the forecast of an on-going aging process of a multi-component system is still challenging. For most complex multi-component systems, it is difficult to obtain enough system-level lifetime data due to the restriction on the trial cost, the limitation of the observation, the very low degradation rate, and so on. An alternative method is to estimate degradation at the component level, leveraging the fact that the component-level degradation is closely associated with the aging of the whole system. The association is defined by the structural function [6], which can be represented by the reliability block diagram [7]. Existing studies, including but not limited to [6,7], neglect the correlations between the components. However, the failure of an individual component usually leads to a load redistribution to other normal components in a complex system, influencing the degradation among components. Therefore, ignoring the correlation may cause unknown risk. There are no formal rules to deal with the interaction of the degradations among connected components [8].

The network approach is widely used to model the spreading dynamics of epidemics and information in society [9,10,11,12,13], and such spreading dynamics resemble the degradation propagation in a complex system. The network approach has demonstrated its advantage in modeling the systems with multiple correlated components [14,15] such as, an electrical circuit with multiple electronic components, a mechanical system involving multiple parts, a living consisting of multiple organs [16,17,18,19,20,21,22,23], and many other.

By combining the network approach, this study develops a MaxEnt-based reliability method for general multi-component systems. The basic idea is to represent the entropy of the system as a function of the hazard rate functions of the participating components. The connectivity of the components in the network can subsequently be recast to an equivalent reliability block diagram of the system. In particular, non-repairable and repairable models are focused to motivate the development of the proposed method. The former one represents a network with multiple inter-connected components where the components only undergo degradation process. The latter one allows for the recovery or replacement of failed components, by which the forecast of an on-going aging-recovering process is demonstrated. To study the degradation propagation, the failure of one component alters the hazard rate function of neighboring components in both models. By incorporating the reliability block diagram, the components are hierarchically organized in a parallel-series diagram. The statistical moments are used in the macroscopic model to reduce the inherent noise in early-stage data. Furthermore, under the assumption of a homogeneous hazard rate, the one-shot type of data can be transformed to equivalent moment data with the reliability block diagram.

This paper is organized as follows. In Section 2, the multi-component system is briefly reviewed. In Section 3, the microscopic model for the non-repairable system is developed. The MaxEnt is used to infer the (inhomogeneous and homogeneous) hazard rates of the components with the different topologies of the network. In Section 4, the reliability block diagram is employed as a tool to aggregate different types of information. In Section 5, the microscopic model for repairable systems is discussed in detail. The repairable-component model of the Watts–Strogatz small world [24] is adopted to demonstrate the proposed method. Different limitations of accessible information, such as the local observation and the one-shot observation, are taken into account.

## 2. Modeling the Degradation and the Recovery Processes

In this paper, the multi-component systems are modeled by the networks, where nodes denote the components. Each component has two possible states, namely, the normal state and failed state.

The propagation of degradation is driven by one or more failed components in the system. The degradation process of a component is triggered by a failed neighboring component with the transition rate x(t), where *t* is the duration that the component connects with at least one failed component. Its remaining lifetime *T* is a random variable with a probability Prob(T>t)=F(t). For a normal component connected to more than one failed component, the transition rate is assumed to be the same. The transition rate function is defined by:

(1) $$x\left(t\right)=-\frac{d\ln F(t)}{dt},$$

which is also called the hazard rate function in reliability theory. With the degradation process defined for an individual component, the joint distributions of all the components’ lifetimes are directly constructed by the hazard functions.

The repairable-component model is built by adding the recovery process. Similar to the degradation process, the recovery time $\tilde{T}$ is assumed to be a random variable with the cumulative distribution $\mathrm{Prob}\left(\tilde{T}>t\right)=R\left(t\right)$. The recovery rate function is defined by:

(2) $$y\left(t\right)=-\frac{d\ln R(t)}{dt}.$$

Recovered components are assumed to undergo further degradation.

## 3. The Non-Repairable System

The standard MaxEnt provides a method to construct the most probable distribution with linear constraints, e.g., moment constraints, or convex constraints [25]. In practice, the small number of constraints may lead to an imprecise inference. For example, for a two-dimensional distribution, if the constraints are the first moments of the two random variables, the standard MaxEnt only provides an uncorrelated distribution, since the first moments do not contain information of correlations. The construction of correlated distributions requires more constraints, which raises a higher requirement of observation.

To reduce the requirement of available information, an alternative way is proposed by combining the MaxEnt with the degradation model, which is regarded as prior knowledge and constrains the probability distribution. Namely, the variation is done in a physical subset of the probability distribution functional space. In this section, the variational probability distributions rely on the network structures and the model. A standard MaxEnt with moment constraints is equivalent to the maximum likelihood estimation, while the MaxEnt based on degradation model here is different from the maximum likelihood estimation.

**Figure 1:**
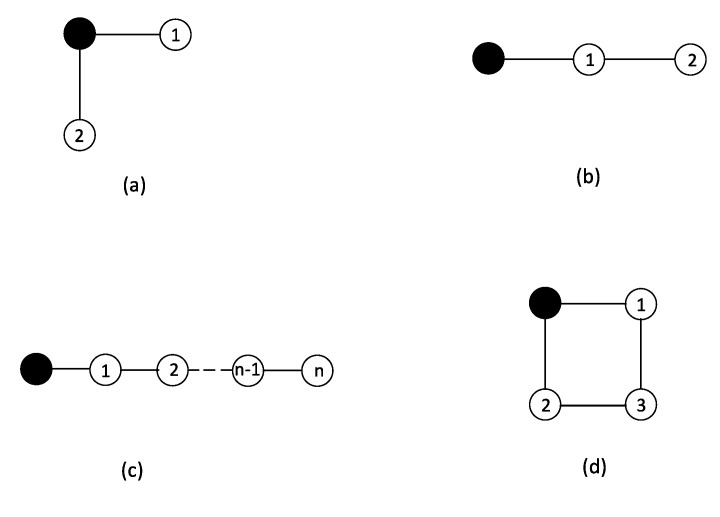
The illustrations of the system networks. (**a**) the double-component model of independent degradation, (**b**) the double-component model of correlated degradation (**c**) the chain-type graph model (**d**) the graph model contains loop. The filled and the numbered circles represent the degradation source and normal components, respectively.

To begin with, the double-component systems as presented in Figure 1a,b, are studied to present the inference of the components’ hazard rates via MaxEnt. The two components are labeled by $C_{1}$ and $C_{2}$ with lifetimes $t_{1}$ and $t_{2}$. The joint probability distribution of lifetimes $p(t_{1},t_{2})$ is associated with the hazard rates $x_{i}$ with i=1,2. The system may degrade in two different possible ways: $C_{1}$ degrades first and $C_{2}$ follows, and the opposite. The joint distribution of the lifetimes is written as:

(3) $$p\left(t_{1},t_{2}\right)\equiv p_{12}\left(t_{1},t_{2}\right)\theta \left(t_{2}-t_{1}\right)+p_{21}\left(t_{1},t_{2}\right)\theta \left(t_{1}-t_{2}\right),$$

where θ denotes the step function. $p_{12},p_{21}$ are the functions depending on the structure of the graph, which will be explicitly defined in different cases. The structure-dependent joint distribution implies the physical subset in which the variation is done.

In the following, the inference of the life time distribution $p(t_{1},t_{2})$ given a different type of information is developed based on the MaxEnt principle. Both the degradation sequence and the lifetimes are considered in the joint distribution (3). The Shannon entropy [26] of the joint distribution is written as:

(4) $$\begin{array}{cc} S[p] & =-\int \int p\left(t_{1},t_{2}\right)\ln p\left(t_{1},t_{2}\right)dt_{1}dt_{2}. \end{array}$$

The linear constraints are:

(5) $$C\left[p\right]=-\sum_{k}\xi_{k}\int \int f_{k}\left(t_{1},t_{2}\right)p\left(t_{1},t_{2}\right)dt_{1}dt_{2},$$

where $\xi_{k}$s are the Lagrange multipliers corresponding to the averages of $f_{k}$ with k=1,2,⋯, and the averages are either the moments or the correlations of the components’ lifetimes. These constraints are the same with that considered in the standard MaxEnt.

The most probable probability distribution is obtained through maximizing the entropy with the constraints:

(6) $$\frac{\delta (S+C)}{\delta p}=0,$$

which also gives the most probable hazard rate.

### 3.1. MaxEnt for Double-Component Non-Repairable Model: Independent Degradation

Figure 1a shows independent degradations of the two components. The joint probability distribution of lifetimes $p\left(t_{1},t_{2}\right)=p_{1}\left(t_{1}\right)p_{2}\left(t_{2}\right)$ is determined by:

(7) $$p\left(t_{1},t_{2}\right)=p_{1}\left(t_{1}\right)p_{2}\left(t_{2}\right)\equiv x_{1}\left(t_{1}\right)x_{2}\left(t_{2}\right)\exp\left[-X_{1}\left(t_{1}\right)-X_{2}\left(t_{2}\right)\right];$$

with the hazard rate function $x_{i}$ of each component and $X_{i}\left(t\right)=\int_{0}^{t}x_{i}\left(t^{\prime }\right)dt^{\prime }$, i=1,2. In this case, $p_{12}$ is same with $p_{21}$, i.e., $p_{12}=p_{21}=x_{1}\left(t_{1}\right)x_{2}\left(t_{2}\right)\exp\left[-X_{1}\left(t_{1}\right)-X_{2}\left(t_{2}\right)\right]$. The hazard rate function is directly used here, because in general a one-dimensional distribution $p_{i}\left(t\right)$ (defined with t∈[0,∞)) can be expressed as $p_{i}\left(t\right)=x_{i}\left(t\right)\exp\left[-X_{i}\left(t\right)\right]$.

By defining a function $L\left(x_{1},X_{1},x_{2},X_{2},t\right)=-p\ln p-\sum_{k}\xi_{k}f_{k}\left(t_{1},t_{2}\right)p$, Equation (6) is rewritten as $S+C=\int Ldt_{1}dt_{2}$. With Euler–Lagrange equations, it follows from Equation (6) that:

(8) $${\dot{x}}_{i}-x_{i}^{2}+x_{i}\sum_{k}\xi_{k}\int_{0}^{t}h_{i}^{\left(k\right)}x_{\bar{i}}\left(t_{\bar{i}}\right)\exp\left[-X_{\bar{i}}\left(t_{\bar{i}}\right)\right]dt_{\bar{i}}=0,$$

where $i,\bar{i}=1,2$, $i\neq \bar{i}$, and $h_{i}^{\left(k\right)}=\partial_{t_{i}}f_{k}\left(t_{1},t_{2}\right){|}_{t_{i}=t}$.

If taking the average lifetimes ${\bar{t}}_{1},{\bar{t}}_{2}$ as the constraints, *i.e.*, $f_{1}=t_{1},f_{2}=t_{2}$, then the solutions become $x_{1}=1/{\bar{t}}_{1},x_{2}=1/{\bar{t}}_{2}$ for Equation (8).

Note that not all the correlations and the moments can be fused by Equation (8). For example, one considers $f_{1}=t_{1},f_{2}=t_{2},f_{3}=t_{1}t_{2}$ and determines the Lagrange multipliers by these observed values ${\bar{t}}_{1}$, ${\bar{t}}_{2}$, and $\bar{t_{1}t_{2}}$. No solution exists for the Lagrange multipliers $\xi_{k}$ in Equation (8) when ${\bar{t}}_{1}{\bar{t}}_{2}\neq \bar{t_{1}t_{2}}$, because the distribution in Equation (7) implies $t_{1}$ is independent with $t_{2}$ which is conflicted with the available information. To remove such conflict, one could modify the degradation model (i.e., modify the physical subset) or select other constraints, for example, $f_{1}=t_{1},f_{2}=t_{2},f_{3}={\left(t_{1}-t_{2}\right)}^{2}$, and the solution to Equation (6) becomes:

$$p\left(t_{1},t_{2}\right)=\frac{1}{Z}\exp\left[-{\tilde{\xi }}_{1}t_{1}-{\tilde{\xi }}_{2}t_{2}-\xi_{3}\left(t_{1}^{2}+t_{2}^{2}\right)\right],$$

where *Z* is the partition function and $-\partial \ln Z/\partial {\tilde{\xi }}_{1}={\bar{t}}_{1},-\partial \ln Z/\partial {\tilde{\xi }}_{2}={\bar{t}}_{2}$, $-\partial \ln Z/\partial \xi_{3}=\bar{{\left(t_{1}-t_{2}\right)}^{2}}+{\bar{t}}_{1}{\bar{t}}_{2}$ with ${\tilde{\xi }}_{1}=\xi_{1}-2\xi_{3}{\bar{t}}_{2},{\tilde{\xi }}_{2}=\xi_{2}-2\xi_{3}{\bar{t}}_{1}$.

### 3.2. MaxEnt for Double-Component Non-Repairable Model: Correlated Degradation Case

In Figure 1b, the degradation processes of the two components are correlated. The joint probability distribution of lifetimes is $p\left(t_{1},t_{2}\right)=p_{12}\theta \left(t_{2}-t_{1}\right)$. Combining the degradation-propagation rule with the network structure, $C_{1}$ degrades due to connection with the degradation source and the degradation of $C_{2}$ follows. As the result, $p_{12}$ becomes:

(9) $$\begin{array}{cc} p_{12}= & p_{1}\left(t_{1}\right)p_{2|1}\left(t_{2}|t_{1}\right) \end{array}$$

where $p_{1}=x_{1}\left(t_{1}\right)\exp\left[-X\left(t_{1}\right)\right]$ is marginal probability distribution, and $p_{2|1}\left(t_{2}|t_{1}\right)=x_{2}\left(t_{2}-t_{1}\right)\exp\left[-X_{2}\left(t_{2}-t_{1}\right)\right]\theta \left(t_{2}-t_{1}\right)$ is the conditional probability distribution. $p(t_{1},t_{2})$ is normalized if $\exp\left[-X_{i}\left(0\right)\right]=1,\exp\left[-X_{i}\left(+\infty \right)\right]=0,i=1,2$. The time-dependent hazard rate function implies a non-Markovian degradation process for the correlated systems. The difference between the distribution by Equations (7) and (9) is caused by different network structures.

Equation (6) becomes:

(10) $$\begin{array}{c} {\dot{x}}_{i}-x_{i}^{2}+x_{i}\sum_{k}\xi_{k}\int_{0}^{t}g_{i}^{\left(k\right)}x_{\bar{i}}\left(t^{\prime }\right)\exp\left[-X_{\bar{i}}\left(t^{\prime }\right)\right]dt^{\prime }=0, \end{array}$$

with $i=1,2,\bar{i}\neq i$ and $g_{1}^{\left(k\right)}=\partial_{t}f_{k}\left(t,t+t^{\prime }\right),g_{2}^{\left(k\right)}=\partial_{t}f_{k}\left(t^{\prime },t^{\prime }+t\right)$.

Equation (9) implies that the random variables $t_{1}$ and $t_{2}-t_{1}$ are statistically independent. With observing the average lifetimes ${\bar{t}}_{1},{\bar{t}}_{2}$, the hazard rates are inferred as $x_{1}=1/{\bar{t}}_{1},x_{2}=1/\left({\bar{t}}_{2}-{\bar{t}}_{1}\right)$. In the above two cases, the entropy functions depend on the structure of the graphs, which leads to different dynamics of degradation processes.

## 4. System Hierarchy by Reliability Block Diagram

In this section, the information constraints to the MaxEnt are considered. The lifetime of one component is the summation of the two intervals. One is the shortest lifetime of the neighbor components. The other is the remaining lifetime of the component. The former relates to the path information, and the single-component information for the latter. The reliability block diagram is introduced to classify different types of information constraints.

### 4.1. System-Level Observation and Coarse-Grained Information

The system-level observation is defined in the following way. For an *n*-component system, a failure of system occurs if more than *k* of *n* components degrade. In the reliability theory, such a system is called the ‘k/n system’ [7]. The lifetime of the entire system is the coarse grain of the component-level information. For the models considered in this paper, the lifetimes of k/n systems depend on the degrading path, and the path information is also the coarse-grain information.

**Figure 2:**
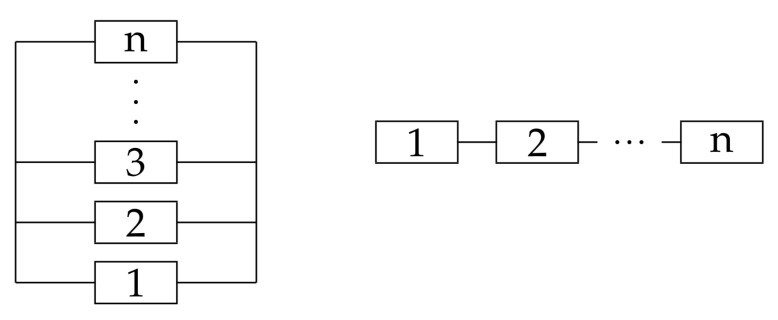
The reliability block diagram of parallel system (**left**) and series system (**right**).

Specifically, the 1/n(n/n) system is called the series (parallel) system, the reliability block diagram of which is shown in Figure 2. In the diagrams, the blocks stand for the components. The diagrams illustrate the relationship between the system-level data and the component-level data.

The reliability block diagram explicitly presents the observed data. As follows, it shows that the reliability block diagram can be reduced according to the degradation rule and the network structure in some particular cases.

### 4.2. Tree-Type Networks

Consider a semi-infinite chain with *n* components, as shown in Figure 1c. From the left side to the right side, the components are labeled by $C_{1},C_{2},\cdots ,C_{n}$. The filled circle stands for a source of degradation. The degradation starts with $C_{1}$ and ends with $C_{n}$. The joint probability distribution of lifetimes is:

(11) $$p\left(t_{1},t_{2},\cdots ,t_{n}\right)=p_{1}\left(t_{1}\right)\prod_{i=2}^{n}p_{i}\left(t_{i}-t_{i-1}\right)\theta \left(t_{i}-t_{i-1}\right).$$

For a parallel system, the average lifetime of $C_{n}$ is observed, which gives the constraint $C=\int p\left(t_{1},t_{2},\cdots ,t_{n}\right)\max\left(t_{1},t_{2},\cdots ,t_{n}\right)\prod_{i=1}^{n}dt_{i}$. According to MaxEnt, the most probable distribution becomes:

(12) $$p\left(t_{1},t_{2},\cdots ,t_{n}\right)=\xi^{n}\exp\left(-\xi t_{n}\right)\prod_{i=2}^{n}\theta \left(t_{i}-t_{i-1}\right),$$

The lifetime distribution of parallel system follows:

(13) $$p\left(t\right)=\frac{\xi^{n}t^{n-1}}{(n-1)!}\exp\left(-\xi t\right).$$

The gamma distribution is retained by MaxEnt with the system-level information.

**Figure 3:**
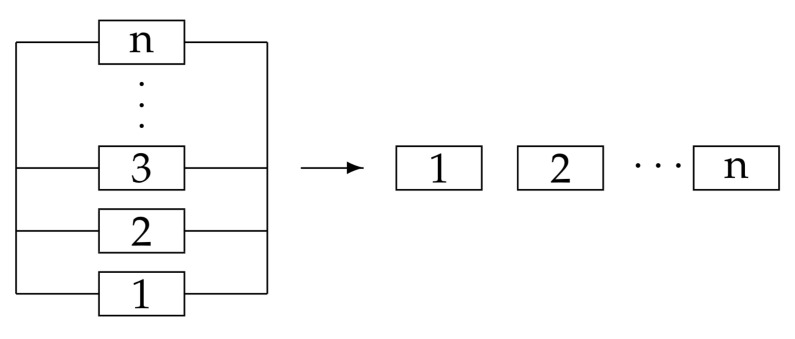
The reduction of the reliability block diagram. The right arrow means that if the *n* components are on one path, the parallel type diagram can be further reduced for tree graphs.

In the chain-type network, the lifetime of $C_{n}$ can be decomposed into the remaining lifetime of each component as $t_{n}=\left(t_{n}-t_{n-1}\right)+\left(t_{n-1}-t_{n-2}\right)+\cdots +t_{1}$. Each interval in the summation is associated with the hazard rate of the corresponding component. This implies that if the *n* components are on one path, the further reduction of the parallel type diagram can be done according to Figure 3. Since the path is unique for any tree graphs, the path information is further reduced to lots of single-component information.

### 4.3. Homogeneous Hazard Assumption

For the degradation propagated on more complex networks, it is difficult to apply the above approach to infer hazard rates of all components, since required information, such as lifetime moments of specific components and subsystems, increases rapidly with the increasing number of components. This information can only be obtained from the observation to system ensemble. However, it is difficult to obtain the ensemble data for a complex system in practice. In particular, it is impossible to make a precise component-dependent inference based on the one-shot degradation data. If the components could be sorted into several classes according to their degrees or other characteristics with negligible difference in the same class, a class-dependent inference is possible to achieve. For example, in the epidemic models, it is usually assumed that all individuals obey the same infection and recovery rates. In this way, it is feasible to infer a homogeneous hazard rate with the one-shot degradation data by MaxEnt.

Under the homogeneous hazard assumption, the variational joint probability distribution of the system in Figure 1c is:

(14) $$p\left(t_{1},t_{2},t_{3},\cdots ,n\right)=\prod_{i=1}^{n}p_{h}\left(t_{i}-t_{i-1}\right)\theta \left(t_{i}-t_{i-1}\right),$$

where $p_{h}$ is the identical remaining lifetime distribution of all the components.

The constraints are the first and the second moment of $t_{n}$: ${\bar{t}}_{n},{\bar{t}}_{n}^{2}$.

(15) $$\begin{array}{cc} C= & -\int p\left(t_{1},t_{2},t_{3},\cdots ,t_{n}\right)\left(\xi_{1}t_{n}+\xi_{2}t_{n}^{2}\right)\prod_{i=1}^{n}dt_{i}, \end{array}$$

where $\xi_{1(2)}$ are the Lagrange multipliers. With $p_{h}=x_{h}\exp\left(-X_{h}\right)$, the MaxEnt by Equation (6) leads to the Euler–Lagrange equation of the hazard rate as:

(16) $${\dot{x}}_{h}-x_{h}^{2}+x_{h}\left({\tilde{\xi }}_{1}+2{\tilde{\xi }}_{2}t\right)=0.$$

The above equation has solution:

(17) $$p_{h}=\frac{1}{Z}\exp\left(-{\tilde{\xi }}_{1}t-\xi_{2}t^{2}\right),$$

where *Z* is normalization constant, ${\tilde{\xi }}_{1}=\xi_{1}+\left(n-1\right){\tilde{\xi }}_{2}{\bar{t}}_{n}/n$. The parameters are determined by:

(18) $$\begin{array}{cc} -\frac{\partial \ln Z}{\partial {\tilde{\xi }}_{1}} & =\frac{{\bar{t}}_{n}}{n}\\ -\frac{\partial \ln Z}{\partial \xi_{2}} & =\frac{\bar{t_{n}^{2}}}{n}-\frac{\left(n-1\right)}{n^{2}}{\bar{t}}_{n}^{2}. \end{array}$$

These results are reduced to the single-component case [2], which coincides with the reduction of the reliability block diagram in Figure 3.

For one-shot observation, it is difficult to infer the component-dependent hazard rate due to the lack of moment information. The homogeneous hazard assumption provides an alternative way to rebuild the joint distribution with the one-shot data.

### 4.4. Loop Networks and Parallel-Series Type Diagram

**Figure 4:**
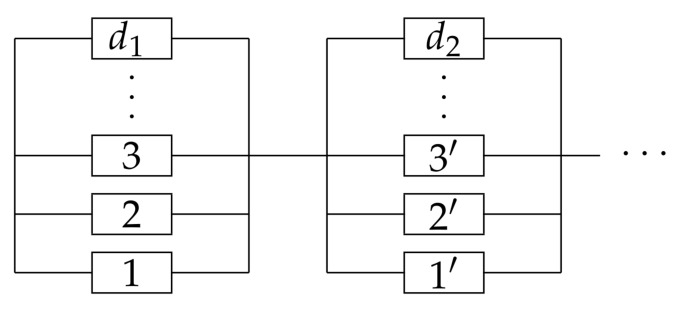
The reliability diagram for parallel-series system.

With a loop structure in a network, the degradation path is not unique, which leads to a parallel-series type diagram. The diagram could not be reduced to single blocks. Consequently, the constraints for the MaxEnt is no longer linear. Assume there are *m* paths from the target component to the source, and the lengths of the paths are denoted by $d_{i},i=1,2,\cdots ,m$. The reliability block diagram is presented in Figure 4.

Take the network in Figure 1d as an example, the components are labeled by $C_{1}$, $C_{2}$, and $C_{3}$. The joint distribution of the lifetime is:

(19) $$\begin{array}{cc} p(t_{1},t_{2},t_{3})= & p_{1}\left(t_{1}\right)p_{2}\left(t_{2}\right)[p_{3}\left(t_{3}-t_{1}\right)\theta \left(t_{3}-t_{1}\right)\theta \left(t_{2}-t_{1}\right)\\ \; & +p_{3}\left(t_{3}-t_{2}\right)\theta \left(t_{3}-t_{2}\right)\theta \left(t_{1}-t_{2}\right)], \end{array}$$

where $p_{i}\left(t\right)=p_{h}\left(t\right)$ is the lifetime distributions of component $C_{i}$.

**Figure 5:**
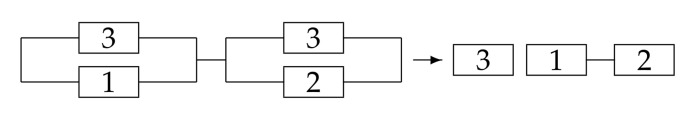
The reliability block diagram and its reduction for a parallel-series structure.

The constraints, for example, are the first-order moments for each individual component, namely:

(20) $$\begin{array}{cc} C= & 3\sum_{i=1}^{3}\xi_{i}\int p\left(t_{1},t_{2},t_{3}\right)t_{i}dt_{1}dt_{2}dt_{3}\\ = & -3\xi \int F_{h}dt-3\xi_{3}\int F_{h}^{2}dt \end{array}$$

where $F_{h}=\int_{t}^{\infty }p_{h}\left(t^{\prime }\right)dt^{\prime }$ is the survival probability of the homogeneous distribution $p_{h}$, and $\xi_{1}=\xi_{2},\xi_{3}$ are the Lagrange multipliers with $\xi =\xi_{1}+\xi_{3}$. The factor 3 in Equation (20) is added to simplify the following calculation and note that it does not affect the distribution determined by the MaxEnt. The first term in Equation (20) is the single-component moment, and the second term is the moment for the series structure of two components. These constraints are directly obtained with the reliability block diagram presented in Figure 5.

To see the non-linearity of the constraint in Equation (20), rewrite the entropy explicitly,

(21) $$\begin{array}{cc} S= & -\int p\left(t_{1},t_{2},t_{3}\right)\ln p\left(t_{1},t_{2},t_{3}\right)dt_{1}dt_{2}dt_{3}\\ = & -\int_{0}^{\infty }dt_{1}\int_{t_{1}}^{\infty }dt_{2}\int_{0}^{\infty }d\tau p_{h}\left(t_{1}\right)p_{h2}\left(t_{2}\right)p_{h}\left(\tau \right)\left[\ln p_{h}\left(t_{1}\right)+\ln p_{h}\left(t_{2}\right)+\ln p_{h}\left(\tau \right)\right]\\ \; & -\int_{0}^{\infty }dt_{1}\int_{0}^{t_{1}}dt_{2}\int_{0}^{\infty }d\tau p_{h}\left(t_{1}\right)p_{h2}\left(t_{2}\right)p_{h}\left(\tau \right)\left[\ln p_{h}\left(t_{1}\right)+\ln p_{h}\left(t_{2}\right)+\ln p_{h}\left(\tau \right)\right]\\ = & -3\int p_{h}\ln p_{h}dt, \end{array}$$

which shows that the entropy of the three-dimensional distribution Equation (19) is proportional to that of the one-dimensional distribution $p_{h}$. It follows from Equation (20) that:

(22) $$S+C=3\left[-\int p_{h}\ln p_{h}dt-\xi \int F_{h}dt-\xi_{3}\int F_{h}^{2}dt\right],$$

where the constraint is nonlinear in $p_{h}$, although still linear in *p*. The degradation model converts the linear constraint in high-dimensional distributions to the non-linear constraint in low-dimensional distributions.

From the structure-dependent joint probability distribution by Equation (19) and the constraints by Equation (20), the hazard rate for each component is inferred as:

(23) $$\begin{array}{c} \ddot{u}+\xi_{3}\dot{u}u=0, \end{array}$$

with $u=F_{h}+\xi /\xi_{3}$. The solution is:

(24) $$\begin{array}{c} F_{h}=\frac{\xi -\xi \tanh(\xi t/2)}{\xi +\left(\xi_{3}+\xi \right)\tanh\left(\xi t/2\right)}. \end{array}$$

The above discussion presents the reduction of the reliability block diagram. It is worth mentioning that the reduction depends on the network structure and the rule of degradation propagation.

## 5. The Repairable System

This section motivates to demonstrate the MaxEnt-based reliability theory for the repairable systems. A failed component can return to a normal component through the recovery process. The recovery processes of the components are assumed to be statistically independent of each other. The degradation is similar to that of the non-repairable model.

### 5.1. Double-Component Model

Consider a simple example as presented in Figure 1b. The component $C_{i}$ fails at time $t_{i}$ and recovers at time $\tau_{i}$ (the recovery time is $\tau_{i}-t_{i}$) with i=1,2. Before the first recovery of $C_{1}$, the component $C_{2}$ may fail at least once or not, which are labeled by (1;1) and (0;1), respectively. Here the notation (n;k) means *k* leaves of the hub with *n* leaves failed at least once.

The joint distributions for two situations are written as:

(25) $$\begin{array}{cc} p_{\left(1;1\right)}\left(t_{2}-t_{1},\tau_{1}-t_{1}\right)= & x\left(t_{2}-t_{1}\right)\exp\left[-X\left(t_{2}-t_{1}\right)\right]y_{1}\left(\tau_{1}-t_{1}\right)\\ \; & \exp\left[-Y\left(\tau_{1}-t_{1}\right)\right]\theta \left(t_{2}-t_{1}\right)\theta \left(\tau_{1}-t_{2}\right),\\ p_{\left(0;1\right)}\left(\tau_{1}-t_{1}\right)= & y\left(\tau_{1}-t_{1}\right)\exp\left[-Y\left(\tau_{1}-t_{1}\right)\right]\theta \left(\tau_{1}-t_{1}\right), \end{array}$$

where the hazard (recovery) rate is *x*(*y*). The corresponding probabilities are obtained as:

(26) $$\begin{array}{cc} P_{\left(1;1\right)} & =\int_{0}^{\infty }\exp\left(-Y\right)x\exp\left(-X\right)dt,\\ P_{\left(0;1\right)} & =\int_{0}^{\infty }y\exp\left(-Y\right)\exp\left(-X\right)dt=1-P_{\left(1;1\right)}. \end{array}$$

Different number of variables in Equation (25) for the two distributions leads to divergent entropy of the joint distribution. In this situation, one could estimate the most probable distributions through maximizing the entropies of the following distributions:

(27) $$\begin{array}{cc} \; & q(t)=y\exp(-Y),\\ \; & p\left(t\right)=\exp\left(-Y\right)x\exp\left(-X\right)/P_{\left(1;1\right)}\equiv \tilde{x}\exp\left(-\tilde{X}\right), \end{array}$$

where *p* is the distribution of the series interval from $C_{1}$ to $C_{2}$, and $\tilde{x}$ is the effective hazard rate.

Based on the average recovery time and series interval obtained from the moments of one-shot data, the hazard and recovery rates are directly inferred from

(28) $$\begin{array}{cc} \delta & [-\int q(t)\ln q(t)dt-\int p(t)\ln p(t)dt\\ \; & -\sum_{k}\left[\xi_{k}\int q\left(t\right)t^{k}dt+{\tilde{\xi }}_{k}\int p\left(t\right)t^{k}dt\right]]=0. \end{array}$$

Particularly for constraints of first and second moments, MaxEnt gives the most probable distribution as:

(29) $$\begin{array}{cc} q(t) & =\frac{1}{Z}\exp\left(-\xi_{1}t-\xi_{2}t^{2}\right),\\ p(t) & =\frac{1}{\tilde{Z}}\exp\left(-{\tilde{\xi }}_{1}t-{\tilde{\xi }}_{2}t^{2}\right), \end{array}$$

where $Z,\tilde{Z}$ are normalization constants.

The bare hazard rate and the ratio of degradation are determined:

(30) $$\begin{array}{cc} \; & p_{\left(1;1\right)}=\frac{1}{\int p(t)\exp(Y)dt}\\ \; & x\exp\left(-X\right)=p_{\left(1;1\right)}\tilde{x}\exp\left(-\tilde{X}+Y\right). \end{array}$$

The ratio of degradation is associated with the recovery duration and the series interval.

### 5.2. Degradation Propagation on Star Graph and Complex Networks

Since in the early stage of a degradation process on a low-clustering-coefficient network, the sub-graph consisting of the failed nodes is usually in a star type, namely most nodes fail due to the connection to one single failed node. As follows, the degradation on a star graph is studied.

A graph is called a star graph if there is one specific node (called ‘hub’) links with all other nodes (called ‘leaves’) and there are no links between the leaves. Consider a star graph with the hub labeled by 0 and the leaves 1,2,⋯,k. Without loss of generality assuming $t_{0}=0$, the detailed joint probability density function $p_{\left(n;k\right)}\left(\tau_{0},t_{1},t_{2},\cdots ,t_{n}\right)$ that *n* of *k* failed is explicitly written as:

(31) $$\begin{array}{cc} p_{\left(n;k\right)}= & y\left(\tau_{0}\right)\exp\left[-\left(k-n\right)X\left(\tau_{0}\right)-Y\left(\tau_{0}\right)\right]\\ \; & \times \prod_{i=1}^{n}x\left(t_{i}\right)\exp\left[-X\left(t_{i}\right)\right]\frac{n!}{(k-n)!n!}, \end{array}$$

with the corresponding probability:

(32) $$\begin{array}{cc} P_{\left(n;k\right)}= & \int p_{\left(n;k\right)}d\tau_{0}\prod_{i=1}^{n}dt_{i}\\ = & \frac{n!}{(k-n)!n!}\int_{0}^{\infty }{\left(1-e^{-X}\right)}^{n}e^{-(k-n)X}qdt. \end{array}$$

It is easy to verify the normalized condition $\sum_{n=0}^{k}P_{\left(n;k\right)}=1$, and the expected number of the components failed at least once:

(33) $$\sum_{n=0}^{k}nP_{\left(n;k\right)}=kP_{\left(1;1\right)}.$$

The moments of the series interval follow from Equation (31) as:

(34) $$\begin{array}{cc} \int_{0}^{+\infty }p\left(t\right)t^{m}dt\equiv & \frac{{\langle t_{1}^{m}\rangle }_{\left(1|1\right)}}{{\langle 1\rangle }_{\left(1|1\right)}}\\ = & \frac{\sum_{n=1}^{k}{\langle t_{1}^{m}+t_{2}^{m}+\cdots +t_{n}^{m}\rangle }_{\left(n;k\right)}/n}{\sum_{n=1}^{k}{\langle t_{1}^{0}+t_{2}^{0}+\cdots +t_{n}^{0}\rangle }_{\left(n|k\right)}/n}, \end{array}$$

where the average denotes ${\langle \cdots \rangle }_{\left(n|k\right)}=\int \cdots p_{\left(n;k\right)}d\tau_{0}\prod_{i=1}^{n}dt_{i}$. Equation (34) does not depend on the leave number *k*, and gives a reliable estimation of moments based on the data of the sampling nodes. The dynamical parameter estimation and network structure estimation, thus, become separated in this way.

### 5.3. Illustration of the Method

**Figure 6:**
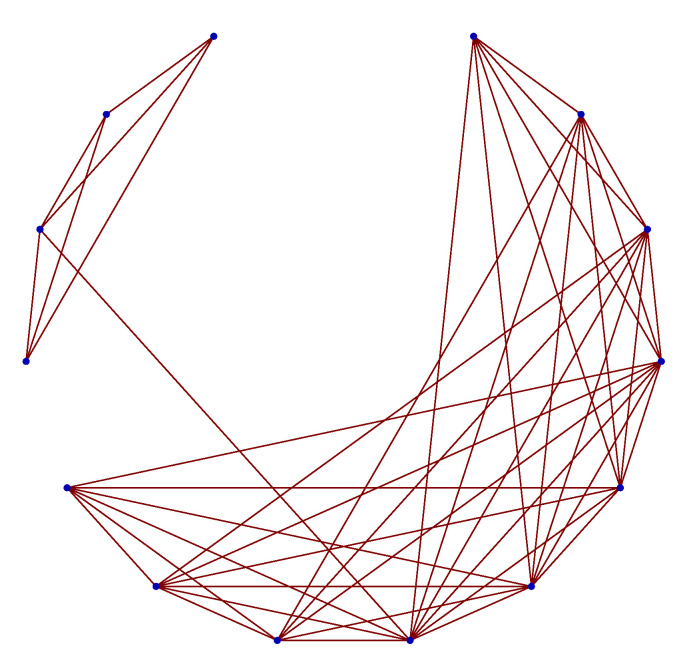
The sub-graph of the generated Watts-Strogatz small world where 14 nodes are shown for illustration purposes.

The performance of the approach is presented with the simulation of the repairable model on 10,000-node Watts–Strogatz small world [24] with the reconnected probability p=0.05 and the degree K=12. The sub-graph of the generated Watts–Strogatz small world is presented in Figure 6 for illustration. To clearly demonstrate the connection 14 nodes are shown.

The hazard rate and the recovery rate are set to be exponential distributions:

(35) $$\begin{array}{cc} \; & x_{\mathrm{sim}}\exp\left(-X_{\mathrm{sim}}\right)\propto \exp\left(-\alpha_{s}t-\beta_{s}t^{2}\right),\\ \; & y_{\mathrm{sim}}\exp\left(-Y_{\mathrm{sim}}\right)\propto \exp\left(-\alpha_{r}t-\beta_{r}t^{2}\right), \end{array}$$

with t>0 In the simulation, the parameters are chosen as $\alpha_{s}=-2$, $\beta_{s}=-1/3$, $\alpha_{r}=1/4$. Here for simplicity, the constant recovery rate is chosen with $\beta_{r}=0$.

**Figure 7:**
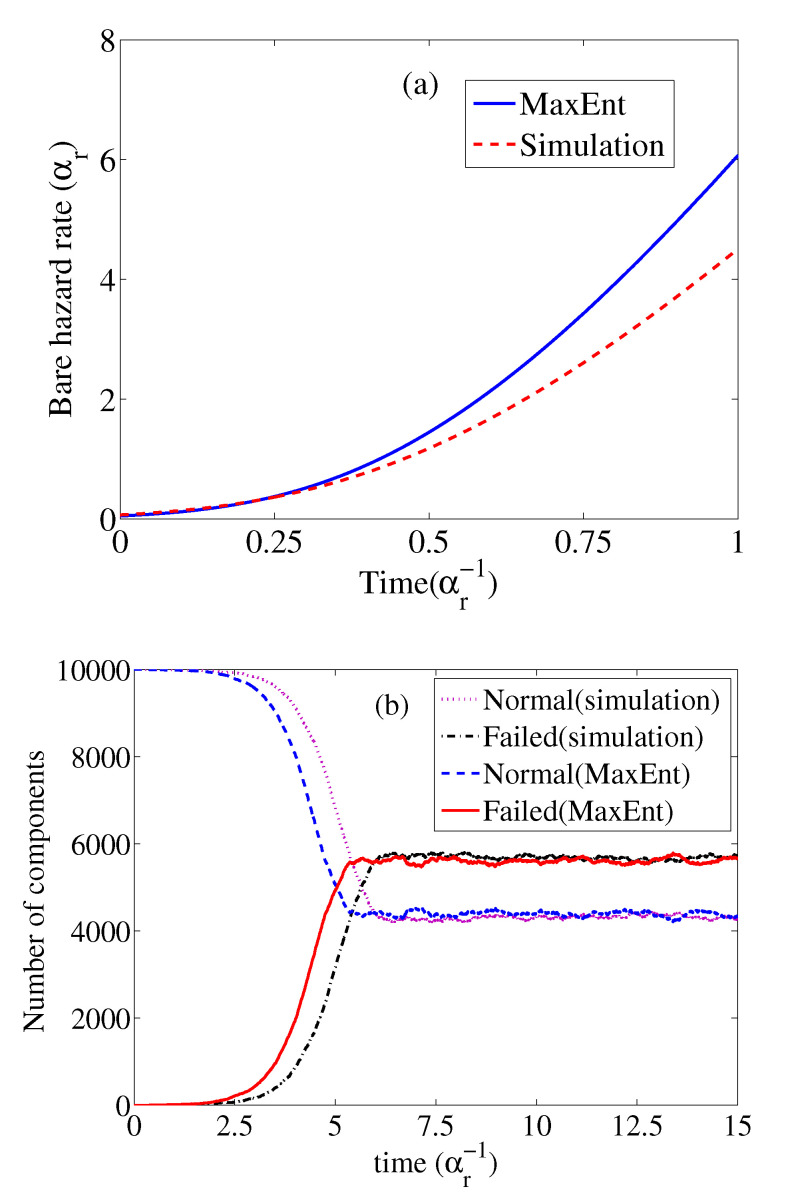
The performance of the MaxEnt method. (**a**) The inference of the bare hazard rate. (**b**) The forecast of the number of normal and failed components by MaxEnt. The parameters of simulation are $\alpha_{s}=-2,\beta_{s}=-1/3,\alpha_{r}=1/4,\beta_{r}=0$. The simulation result shows that the total spreading last about $T=15(\alpha_{r}^{-1})$, the data with an initial $1.5(\alpha_{r}^{-1})$. The data of 27 single-component samples are used to estimate the moments.

The constraints are the first and second moments of the series interval and the first moment of recovery time. With the reliability block diagram, the observed component is chosen to ensure that only single-component data is obtained. The results are presented in Figure 7.

For the system-level models, the reliable estimation of the model parameters depends on sufficient system-level survey data. For example, in the epidemics [27], the numbers of susceptible, infected, and recovered individuals are needed at different time points to estimate the model parameters. The counts are noisy due to the false alarm, test capacity, and latency [28]. It is different with the approach in [27] that the proposed approach can deal with the component-level data.

## 6. Conclusions and Discussion

In this work, a novel MaxEnt-based approach of multi-component systems was proposed to assess the reliability of non-repairable and repairable systems. The developed approach provides a rational way to estimate hazard rates of a system consisting of correlated degrading components. Combined with the reliability block diagram, the one-shot type of data can be used for the estimation. The case study shows that the developed approach can yield reliable results with limited and noisy data at the early stage.

**Figure 8:**
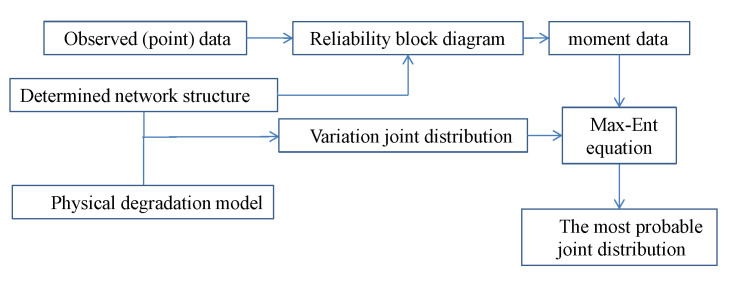
The block diagram of proposed method.

The application of the approach involves the following steps in general as presented in Figure 8. (1) Form a network with nodes representing the multi-component system, (2) build the variational joint distribution based on the network, (3) collect the observed lifetime (recovery duration) data of the components as testable information, (4) process the observed data according to the reliability block diagram and calculate the moments, and (5) maximize the entropy of the variational joint distribution with the moment constraints. For many artificial systems, the network structure is usually known, and the network can be constructed accordingly among the components. For the systems with an unknown structure, the inference of network structure is also needed. Such inference is not the subject of this paper. Relevant discussions can be found in [29,30] for network modeling. Combination the inference of network and dynamics will be studied in future work.

## Data Availability

Not applicable.
